# Designing water vapor fuelled brine-silk cocoon protein bio-battery for a self-lighting kettle and water-vapor panels

**DOI:** 10.1038/s41598-022-18211-x

**Published:** 2022-08-17

**Authors:** Himanshi Jangir, Mainak Das

**Affiliations:** 1 NanoScience Technology Center, University of Central Florida, Orlando, Fl 32826 USA; 2 Design Department, Indian Institute of Technology Kanpur, Kanpur, UP 208016 India

**Keywords:** Chemical engineering, Biological techniques, Biomaterials, Materials for devices, Soft materials

## Abstract

Water vapor increases the electrical conductivity of silk cocoons, human hair, jute, and corn silk. This phenomenon is unclear. In the present study, XPS analysis of cocoons showed that water vapor reduces the surface presence of low-energy carbon species (C–C, C–H). In contrast, electron-dense, high-energy carbon species (C–N, C=C, C=O) remained unchanged, possibly enhancing surface charge hopping. While water vapor improves the conduction, the deficiency of charge carrier diminishes the effect. We increase the charge carrier by soaking the cocoon in an aqueous solution of common salt (NaCl) to amplify the current. Salt treatment followed by 2-min exposure to water vapor results in a sharp upward spike in the current (3.6 ± 1.07 mA, n = 12; mean ± SE) from the baseline (0.06 ± 0.02 mA, n = 12). After 1 h, it maintains an average value of 0.39 ± 0.12 mA; n = 12, indicating an upward shift in the baseline. Every time the cocoon charges with water vapor, the next charging cycle initiates after the cocoon dries up. Inspired by the cocoon ecology, we demonstrate an alternating 'water vapor–dry air' cycle for rapid charging and discharging of the cocoon battery. Finally, we designed a prototype of a self-lighting kettle and water–vapor panels for futuristic homes using a 'brine-silk cocoon protein bio-battery,' where moist waste heat generates electricity.

## Introduction

Membrane proteins present in the cells are bio-inspiration for the bio fabrication of futuristic energy harvesting and storage devices^1,2^. The inherent challenges to designing and fabricating such devices are the complexity of isolation of such proteins and their short shelf life. We approached the problem with a naturally occurring robust protein-membrane silk cocoon to anticipate these challenges^3^.

A silk cocoon is a protein membrane formed by lepidopteran insects. A lepidopteran insect has a typical four-stage lifecycle: egg, larvae, pupae, and adult moth. An adult moth lays the eggs, and upon hatching, the larvae emerge. The larvae voraciously eat on the plant leaves and copiously secrete a viscous salivary fluid rich in protein, termed silk. It spun this silky fluid around its own body, thus forming a close protective cocoon chamber. It marks the beginning of the pupal phase (dormant or diapause phase) in an insect's life. This phase varies from 21 days to 9 months in certain species of worm found in the temperate regions of the world. Once this self-induced dormancy is complete, an adult moth emerges out of the cocoon. This whole process is a metamorphosis^4,5^.

A cocoon is an incubator that maintains an ambient temperature and selectively facilitates the diffusion of carbon dioxide outside the cocoon^5^. It protects the developing pupae from direct sunlight, rain, wind, and green-house effect^6–8^. The decrease in pore size from outside to inside the silk protein membrane prevents water from seeping inside the silk cocoon, making it a water-proof membrane^9–11^. The harsh UV rays are absorbed on the cocoon surface by antioxidant UV protectant compounds present on the outer surface of the silk cocoon^6–8,12^. The silk cocoon senses gravity through its soft magnetic features, thus supporting the healthy development of the pupae^13^.

Earlier it has been proposed that the temperature regulation in silk cocoons is a thermo-electric phenomenon encompassing Seebeck, Peltier, and Thomson-like effects^5,10–12,14–16^. The structural asymmetry between silk cocoons' outer and inner surfaces further strengthens this idea^5^. We found out that when the silk cocoon membrane is placed between two electrodes and exposed to water vapor, it generates current to power LEDs. LED stops glowing when we switch off the water vapor. A dry cocoon fails to generate discernible current when exposed to moisture-free air. A moist silk cocoon can hold charge briefly like a capacitor when charged with a DC source^10,11^. The charge carriers within silk cocoons are water-mediated polaron-like charged complexes activated by heat and moisture^16^. One emerging theme of the thermoelectric properties of the cocoon is the need for water molecules in the intermolecular spaces of the protein, unlike any known solid-state thermoelectric materials. So, it is a wet-thermoelectric material^10–12,14–16^. The basic philosophy of the work is to exploit the wet thermoelectric property of silk cocoon to develop a silk protein-energy device that could supply power for an extended time even after stopping the water vapor and functions in a vast regime of weather.

So, to develop our working hypothesis to fabricate a silk protein-energy device, we first consider the two prerequisites essential for silk cocoon electricity: water and heat. The water activates the backbones and side chains of the silk cocoon protein. At the same time, the heat perturbs the hydrogen bond network of water surrounding the protein and causes the flow of water-mediated polaron-like charged complexes^16^. So, we hypothesize that adding an extra ionic charge carrier in the silk-water complex will increase the electrical output for an extended period and may alter the silk protein surface properties. We chose sodium chloride (NaCl) as the charge carrier since the proteins in the biological system function in a brine-rich extracellular fluid and are the most abundant salts in the earth's water reserve.

## Materials and methods

### Silk cocoon

**Figure 1:**
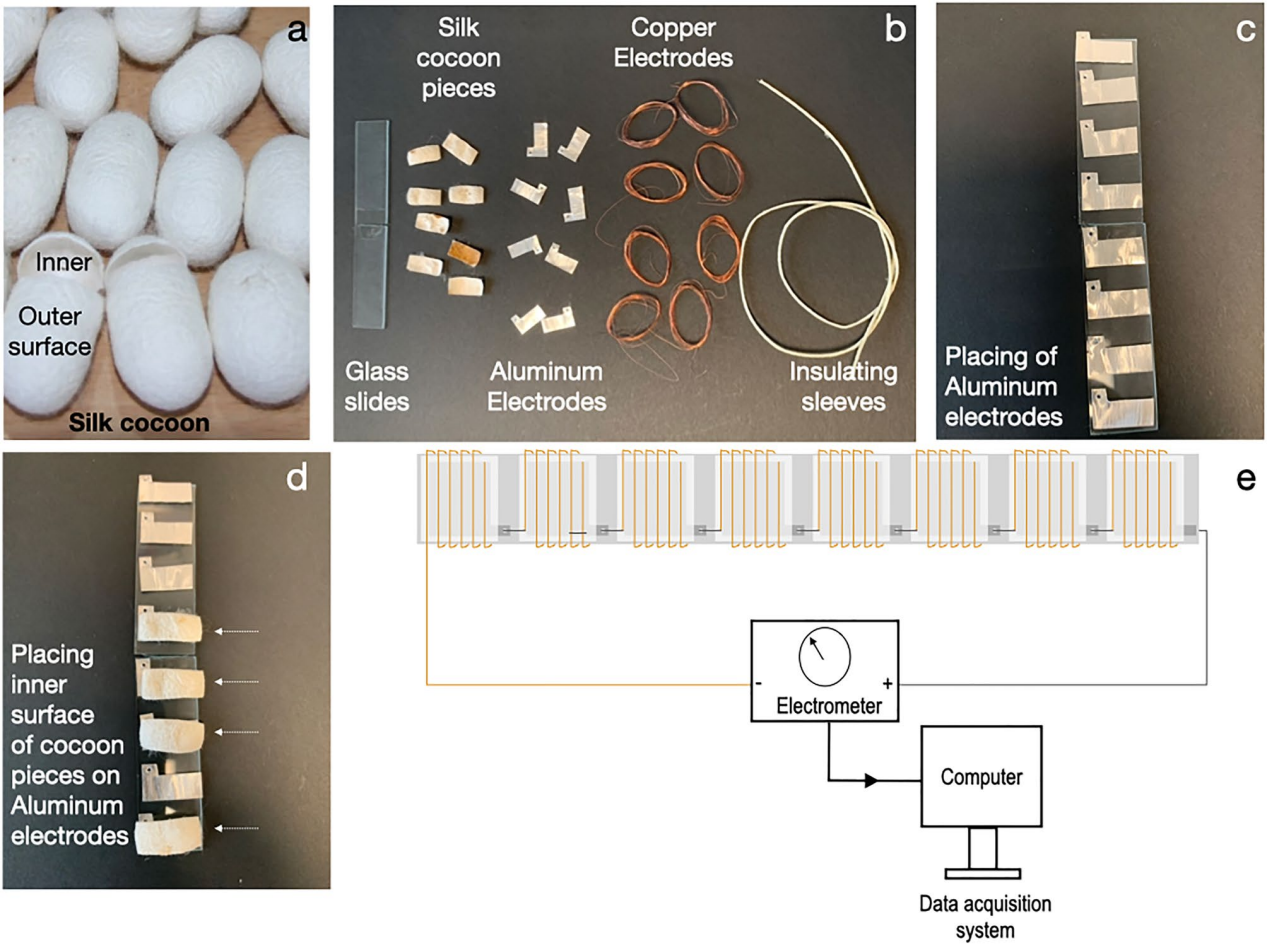
Materials for silk cocoon biobattery. (**a**) Raw Bombyx mori silk cocoons. Cut open the cocoon showing the inner and outer surface of the cocoon with a distinctly different texture. (**b**) All the components for silk cocoon battery. Two glass slides glued together. Silk cocoon pieces with a dimension of 1.1 cm * 2.5 cm. 32 Gauge aluminium electrode cut from aluminium sheet. High purity 1.12 m copper wires (32 AWG) for copper electrodes. Insulating sleeves. (**c**) Placing the aluminium electrodes on the glass slide. (**d**) Placing the inner surface of cocoon pieces facing the aluminium electrode (shown in dotted arrows). (**e**) The device connects to the electrometer and records data in the acquisition system. In the supplementary section, we have a video (Video DIY) explaining the process of making the water vapor-fuelled brine-silk cocoon protein bio-battery.

We procured the Bombyx mori silk cocoons from Orissa and Chhattisgarh in India. We dried the cocoon in sunlight to ensure that no pupae survived inside and then blow-dry to remove any dust and store them in the wooden cabinet for further use (Fig. 1a,b). In the supplementary section, we have a video (Video DIY) explaining the process of making the water vapor-fuelled brine-silk cocoon protein bio-battery.

### Soaking silk cocoon in NaCl

We saturated the silk cocoon pieces (Fig. 1b) in an aqueous solution of NaCl. We add 12.5 g of NaCl, and to that, we add 25 ml of double-distilled water. In each of these solutions, we add sixteen pieces of silk cocoon and leave them soaked for 24 h. The supplementary material (Table 1 of supplementary material) discusses the dose optimization for NaCl. We prepare the NaCl-free devices by wetting the silk cocoon pieces in double-distilled water for 24 h. For comparison with other salts, we prepared KCl devices the way we designed NaCl devices (refer to supplementary section Table 2 of supplementary material) for a comparison of NaCl versus KCl devices' electrical values to distilled water-treated devices).

### Design of silk cocoon energy devices

**Figure 2:**
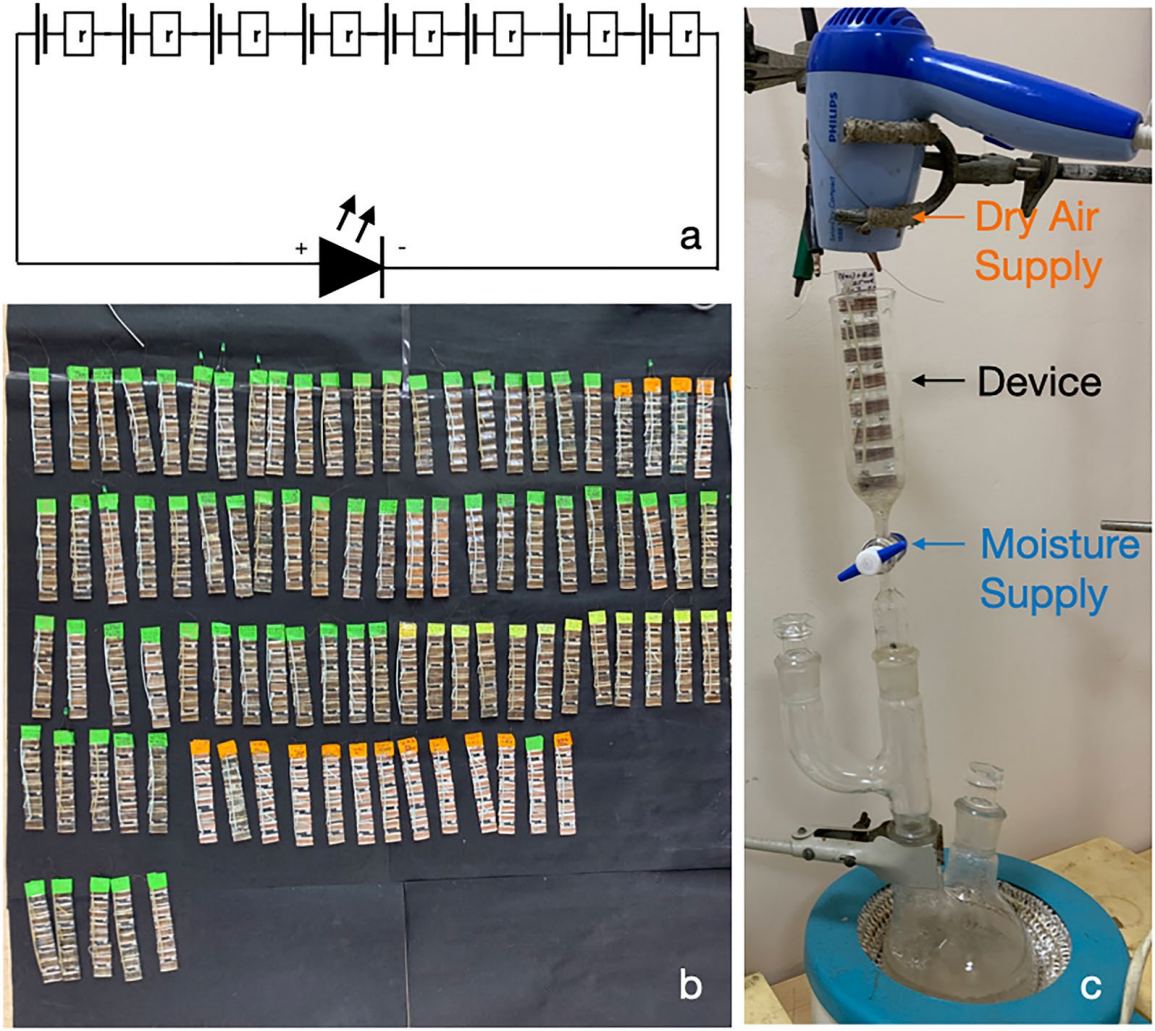
Procedure for making silk cocoon biobattery. (**a**) Eight silk electrochemical cells connected in a series circuit. (**b**) The array of silk cocoon bio-battery. (**c**) Boiling water from the round bottom flask supplies water vapor. The temperature at the surface of the device is around 55–60°. (**d**) A hairdryer blows dry air to evaporate the water from the device. The devices get 2 min of water vapor exposure in all the experiments.

In Figs. 1 and 2, we have shown all the materials and procedures for making silk cocoon bio-battery. We take eight pieces of Bombyx mori cocoon to make a single device. We empirically reach the use of eight pieces of the cocoon after multiple trials during our last 12 years of research. Earlier, we made devices with one-piece, two, four, and eight pieces of the cocoon. Numerous charging-discharging cycles indicate that the eight-piece devices perform for a prolonged period from 1 to 6 months if stored properly. Since it is in the technology discovery phase, we opted for using eight pieces of the cocoon to develop the device prototype. To obtain eight pieces of the cocoon, we use four cocoons. From each cocoon, we obtain two pieces for the device. A single device consists of eight individual electrochemical cells connected in series (Fig. 2a). Each electrochemical cell is fabricated by placing an aluminium electrode (2.4 cm * 1 cm) on the inner surface of the particular cocoon piece and then spooling the copper wire along the outer surface of the cocoon; So the cocoon piece remains between aluminium and copper electrodes. We mount the device on a glass slide. We connect the eight electrochemical units in a series circuit, and the copper end (Positive) and the aluminium terminal (Negative) feed the power to a green light-emitting diode (LED). In Fig. 2b,c, we have the picture of the devices and the in-house set-up for water vapor exposure and drying cycles.

### Electrical recordings

We perform all the electrical recordings in Keithley's 5½-digit Model 6517B electrometer/high resistance meter (Keithley Instruments, Inc. 28775, Aurora Road, Cleveland, Ohio, 44139, USA; http://www.keithley.com/company).

### XPS spectra

We took the X-ray photoelectron spectra using the PHI 5000 Versa Prob II, FEI Inc XPS model.

### Statistical analysis

We have presented the average values as mean ± standard error, where n represents the number of devices. We use Prism 9 software for plotting the graphs and the statistical analysis.

## Results

### Electrical properties of the silk bio-battery

**Figure 3:**
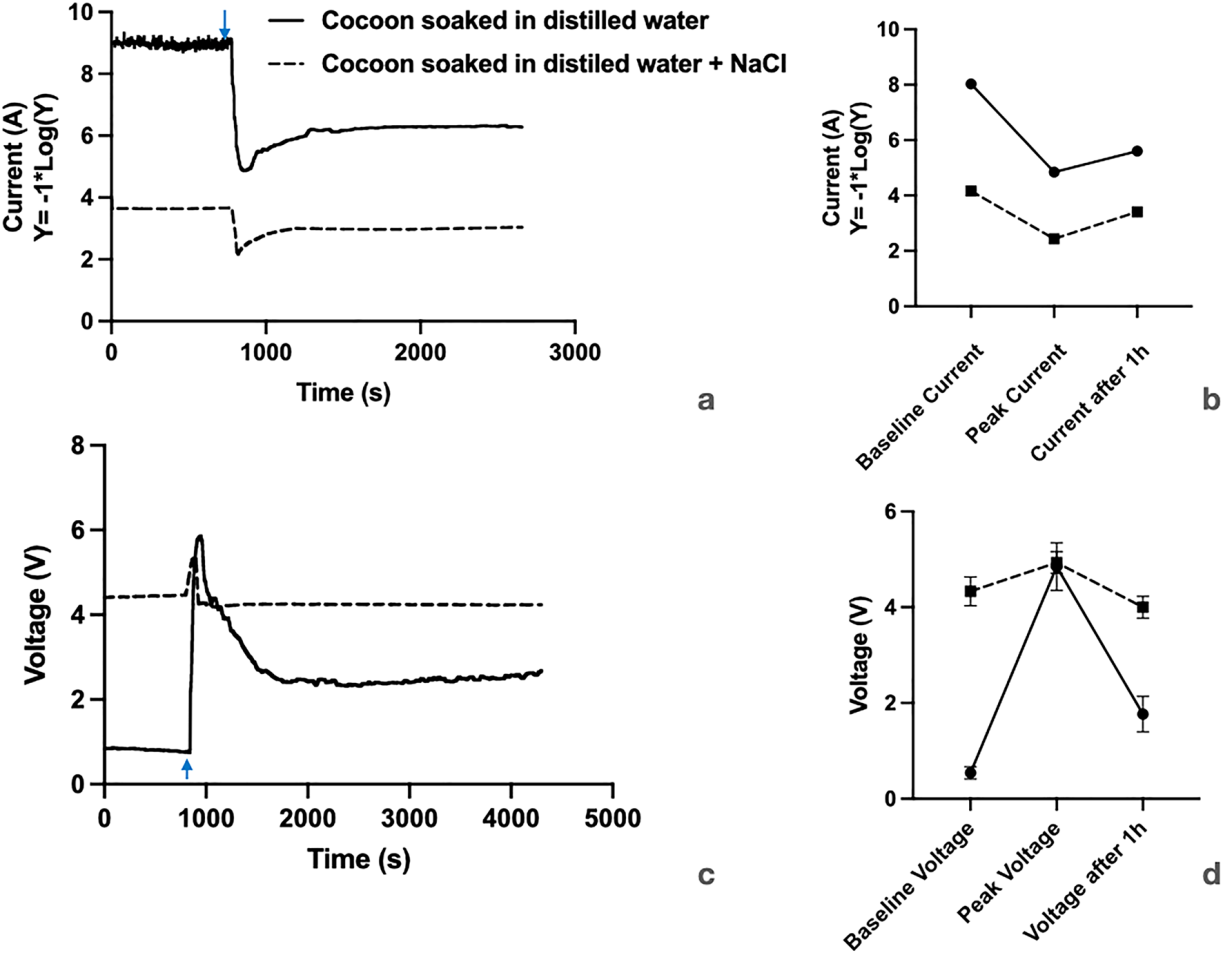
Electrical properties of the silk cocoon devices. (**a**) Comparing a representative current trace from devices prepared from the distilled water-soaked cocoon and NaCl soaked cocoon. The current values are shown in the −1*Log (y) scale. The blue arrow indicates the 2 min time exposure of water vapor. (**b**) Comparing the current values of baseline, peak, and after one h for distilled water soaked and NaCl soaked cocoon devices. (**c**) Comparing a representative voltage trace from devices prepared from the distilled water-soaked cocoon and NaCl soaked cocoon. (**d**) Comparing the voltage values of baseline, peak, and after one h for distilled water soaked and NaCl soaked cocoon devices.

In Fig. 3, we show the basic electrical properties of the silk bio-battery. In Fig. 3a, we have shown two representative traces of current generated by cocoon devices made from distilled water and NaCl soaked cocoons, respectively. The baseline current is higher in the NaCl soaked cocoon device. Soon after the 2 min of water vapor exposure, the current increases sharply from the baseline, and after reaching the peak, it starts to fall. Eventually, the current attains a stable value, higher than the baseline value in both kinds of devices. We next quantified the baseline, peak, and after 1-h current values generated from both types of devices (Fig. 3b). The average baseline current values for distilled water and NaCl soaked devices are 9.33e−06 ± 6.24e−06 mA; n = 6 and 0.06 ± 0.02 mA; n = 12 respectively (average current ± standard error; n = number of devices tested). The average peak current values for distilled water and NaCl soaked devices are 0.014 ± 0.003 mA; n = 6 and 3.63 ± 1.07 mA; n = 12, respectively. So the average peak current value for NaCl soaked device is approximately 259 times more than the distilled water-soaked cocoon device. After 1 h, the average current values for distilled water and NaCl soaked devices are 0.002 ± 0.0001 mA; n = 6 and 0.39 ± 0.10 mA; n = 12 respectively. Comparing the current at baseline and after 1 h for the NaCl device, we find a fivefold increase. Hence we conclude that water vapor exposure charges the membrane. Refer to supplementary section for comparison of NaCl versus KCl devices’ electrical values.

Figure 3c presents the representative voltage traces for cocoon devices made from distilled water, and NaCl soaked cocoons. In both cases, we observe a sharp peak in voltage. The average peak voltage of cocoon devices made from distilled water and NaCl soaked cocoons is 4.8 ± 0.49 V; n = 6 and 4.9 ± 0.22 V; n = 6, respectively; indicating similar peak voltage, unlike the sharp difference in current values (Fig. 3d). The baseline voltage of the distilled water-soaked device is much lower (0.54 ± 0.12 V; n = 6) than the NaCl-soaked device (4.33 ± 0.30 V; n = 6). We observe a much more abrupt change in voltage upon exposure to water vapor in the case of the distilled water-soaked device. The voltage after 1 h in the distilled-water-soaked device versus the NaCl-soaked device is 1.7 ± 0.36 V; n = 6 and 4.0 ± 0.22 V; n = 6, respectively. We observe that the baseline and after 1-h voltages for the NaCl-soaked devices vary very little.

**Figure 4:**
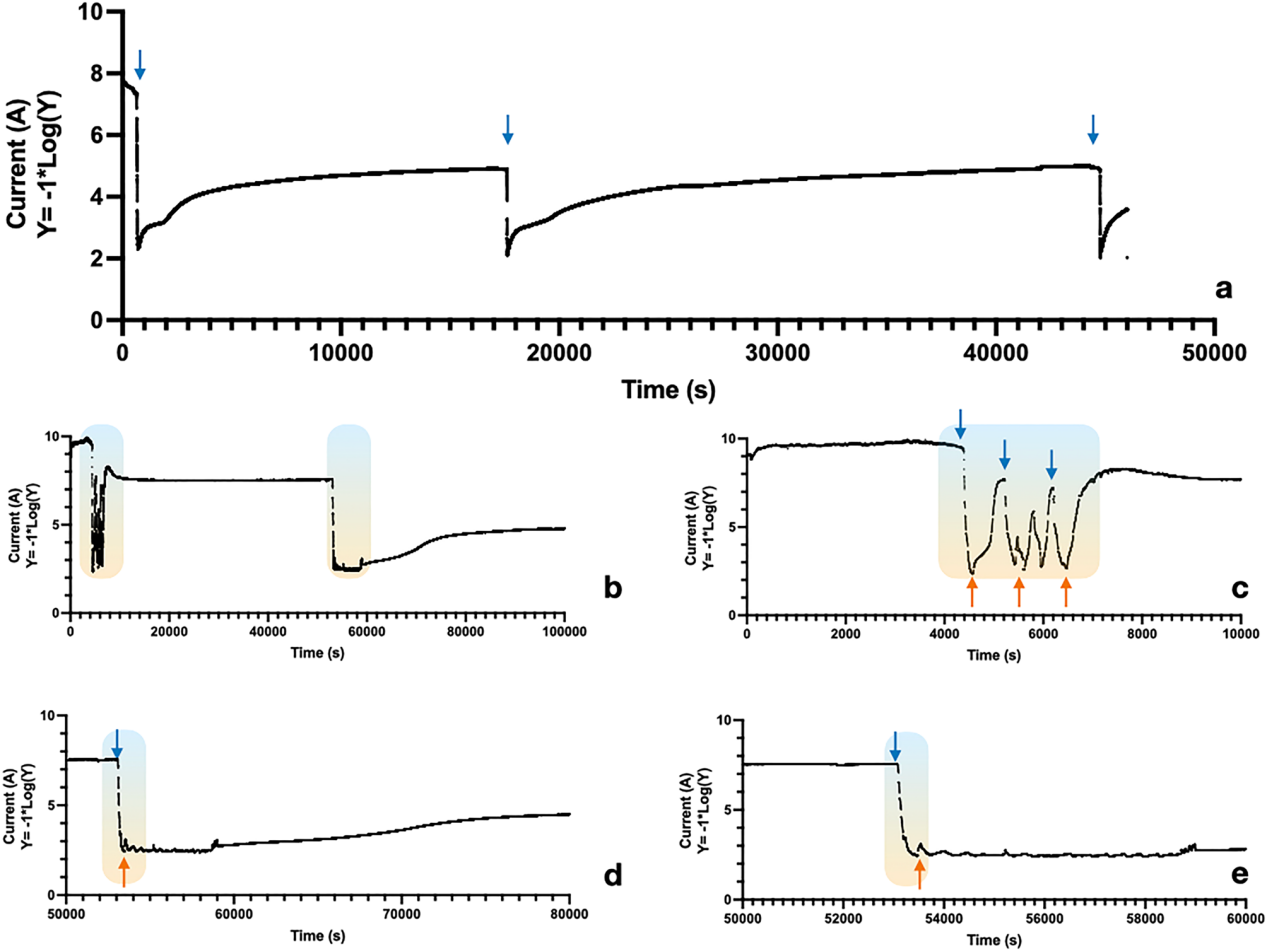
Charging-Discharging features of NaCl-soaked cocoon device. (**a**) Representative current trace for 13 h, with three charging cycles. Blue arrows indicate the time of water vapor exposure. (**b**) Representative current trace for 27.7 h with intermittent water–vapor and rapid drying cycles. The color-shaded zones indicate the cycles. (**c**) The blown-up image of the first colored shaded zone. Thrice this device is charged with water vapor and dried with a blower (orange arrow indicates dry air). (**d**) The blown-up image of the second colored shaded zone. This device is charged once with water vapor and dried with a blower. (**e**) The magnified image on the current trace indicates that the baseline current has increased.

In the next set of experiments, we studied the charging-discharging feature of the NaCl soaked cocoon device. In Fig. 4a, we have presented a representative current trace of the charge–discharge cycle where the device ran for 13 h. At the beginning of the cycle, the device is charged with water vapor and then recharged at two-time points with water vapor, at 4.7 h and 12.5 h, respectively. The device dries up between every charging cycle. We find that as the device cycles, the peak current output increases. The peak current for the three charging points is 4.8 mA, 7.8 mA, and 9.3 mA, respectively. The system needs to dry up before we can recharge the system. To view the effect of rapid drying of the membrane, soon after exposing it to water vapor, we expedite the drying by blowing a stream of dry air for 1 min. Figure 4b consists of a representative current trace, where we have charged the device with water vapor followed by a 1-min drying cycle. We conducted a 27.7 h of recording (Fig. 4b). Drying the device helps to recharge the system immediately, as seen in Fig. 4c. Figure 4d,e show that the baseline current increases markedly after the water vapor and drying cycle.

### Surface analysis of cocoons

**Figure 5:**
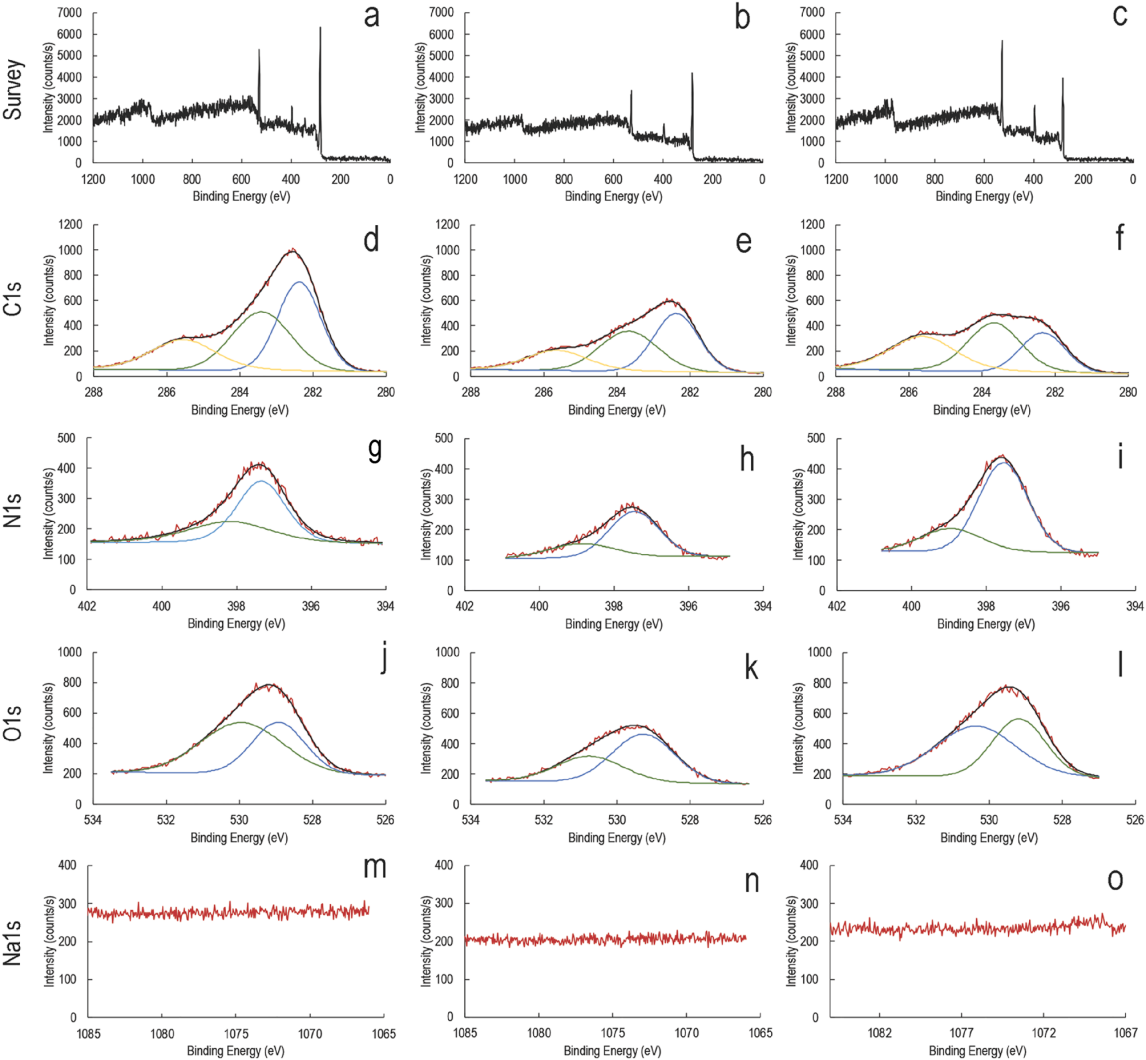
Representative XPS spectrum for dry silk cocoon, silk cocoon soaked in water, silk cocoon soaked in water and exposed to water vapor. Survey spectrum of the dry silk cocoon. Blue, green, and yellow color XPS peaks code lowest to the highest binding energy. The corresponding binding energy values with respective color coding are available in Table 1 (**a**), silk cocoon soaked in water (**b**), silk cocoon soaked in water, and exposed to water vapor (**c**). Comparative C1s spectrum for the dry silk cocoon (**d**), silk cocoon soaked in water (**e**), silk cocoon soaked in water, and exposed to water vapor (**f**). Comparative N1s spectrum for the dry silk cocoon (**g**), silk cocoon soaked in water (**h**), silk cocoon soaked in water, and exposed to water vapor (**i**). Comparative O1s spectrum for the dry silk cocoon (**j**), silk cocoon soaked in water (**k**), silk cocoon soaked in water, and exposed to water vapor (**l**). Comparative Na spectra for the dry silk cocoon (**m**), silk cocoon soaked in water (**n**), silk cocoon soaked in water, and exposed to water vapor (**o**).

**Figure 6:**
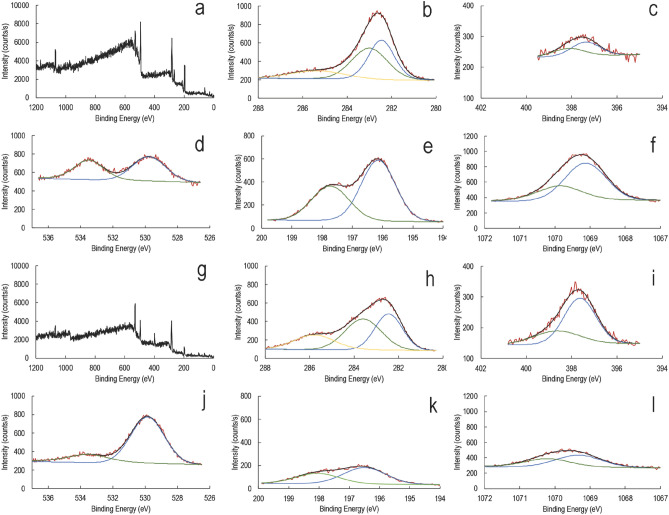
XPS spectrum to observe changes in silk cocoon surface composition upon interaction with NaCl. Blue, green, and yellow color XPS peaks code lowest to the highest binding energy. The corresponding binding energy values with respective color coding are available in Table 1. *XPS for cocoon soaked in aqueous NaCl*, (**a**) Survey spectrum, (**b**) C1s spectrum, (**c**) N1s spectrum, (**d**) O1s spectrum, (**e**) Cl2p spectrum, (**f**) Na1s spectrum; *XPS spectra for cocoon soaked in aqueous NaCl and exposed to water vapor*, (**g**) Survey spectrum, (**h**) C1s spectrum. (**i**) N1s spectrum, (**j**) O1s spectrum, (**k**) Cl2p spectrum, (**l**) Na1s spectrum.

We used X-ray photoelectron spectroscopy (XPS) to analyze the cocoon surfaces, and Figs. 5, 6 and Table 1 summarise the data. As shown in Fig. 5a–c, the overall presence of Carbon, Nitrogen, and Oxygen on the cocoon surface reduces upon wetting (Fig. 5b) and then slightly recovers back upon exposure to water vapor (Fig. 5c). Upon further exploring the narrow spectrum for C1s, in Figs. 5d–f, we observed that the presence of low bond energy species of carbon on the surface reduces upon wetting and further upon exposure to water vapor. Nitrogen and oxygen narrow-spectrum show a similar trend of reducing surface species and their recovery upon exposure to water vapor (Fig. 5g–l). So, as shown in the table, the C:N:O surface composition in dry form is 4:1:2.5. In wet form 2.64:0.55:1.71 and upon water vapor exposure is 3.0:0.99:2.57. The changing C:N:O ratio is evidence of membrane reorganization, where wetting leads to softening of the originally rigid membrane. Exposure to energy-rich water vapor fuels the alignment of electron-rich species like C–N, C=O, and OCNH2 species that act as molecular wires to conduct charge. So, this biological membrane is a programmable matter exhibiting its transformation from poorly conducting to conducting matrix upon interaction with energy-rich water molecules. Figure 5m–o showed the baseline spectra of Na1s.

We next add NaCl to the cocoon to increase the charge carriers. We conducted a detailed XPS analysis (Fig. 6a–l) to understand the surface chemical changes upon introducing NaCl to the silk cocoon. Figure 6a,g show the survey spectra for cocoons soaked in NaCl and NaCl-soaked plus exposed to water vapor. In the NaCl soaked cocoon, the surface composition of carbon reduces, and nitrogen and oxygen increase upon exposure to water vapor, indicating the structural reprogramming of the membrane (Fig. 6b–d,h–j). In narrow spectra of oxygen, a 530 eV peak corresponds to lattice oxygen. While a second peak at 533.5 eV signifies exposed oxides to water, indicating dissociative adsorbed water molecules or surface hydroxyls. As XPS is a surface analysis technique evaluating the elemental composition in less than the top 10 nm of the surface, a drastic reduction in the presence of sodium and chloride shows that these elements percolate in the membrane upon exposure to water vapor (Fig. 6e,f,k,l). The seeping in of the sodium and chloride ions inside the protein framework possibly leads to the formation of conducting bridges, resulting in increased conductance. The basal conductance increases upon adding sodium chloride, but the membrane still maintains its native characteristic features. All the assigned values for the different peaks are in Table 1.

**Table 1:**
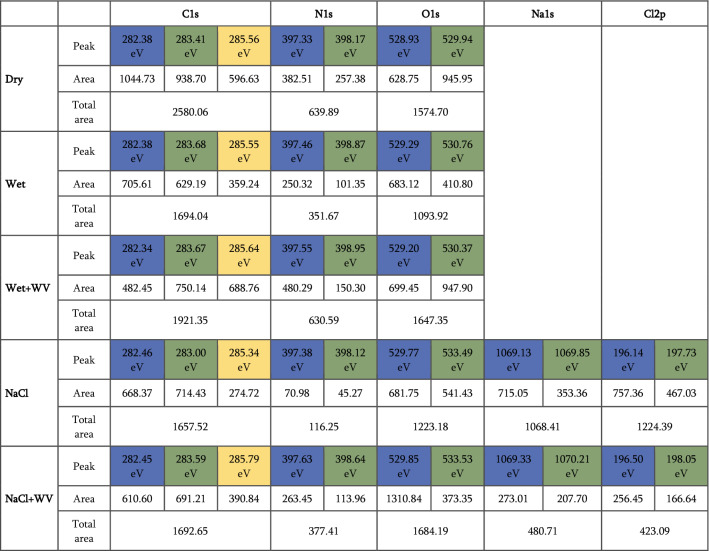
The binding energies from the narrow spectra for each constituting element under different conditions.

WV stands for water vapor. Blue, green, and yellow color XPS peaks code lowest to the highest binding energy. The corresponding binding energy values with respective color coding are available in the Table 1 and tallies with Figs. 5 and 6.

With the 17th-century steam engine discovery by Thomas Newcomen and James Watt, we develop the prototype of a self-lighting kettle and water–vapor panels for futuristic homes using a 'brine-silk cocoon protein bio-battery,' where moist waste heat generates electricity.

**Figure 7:**
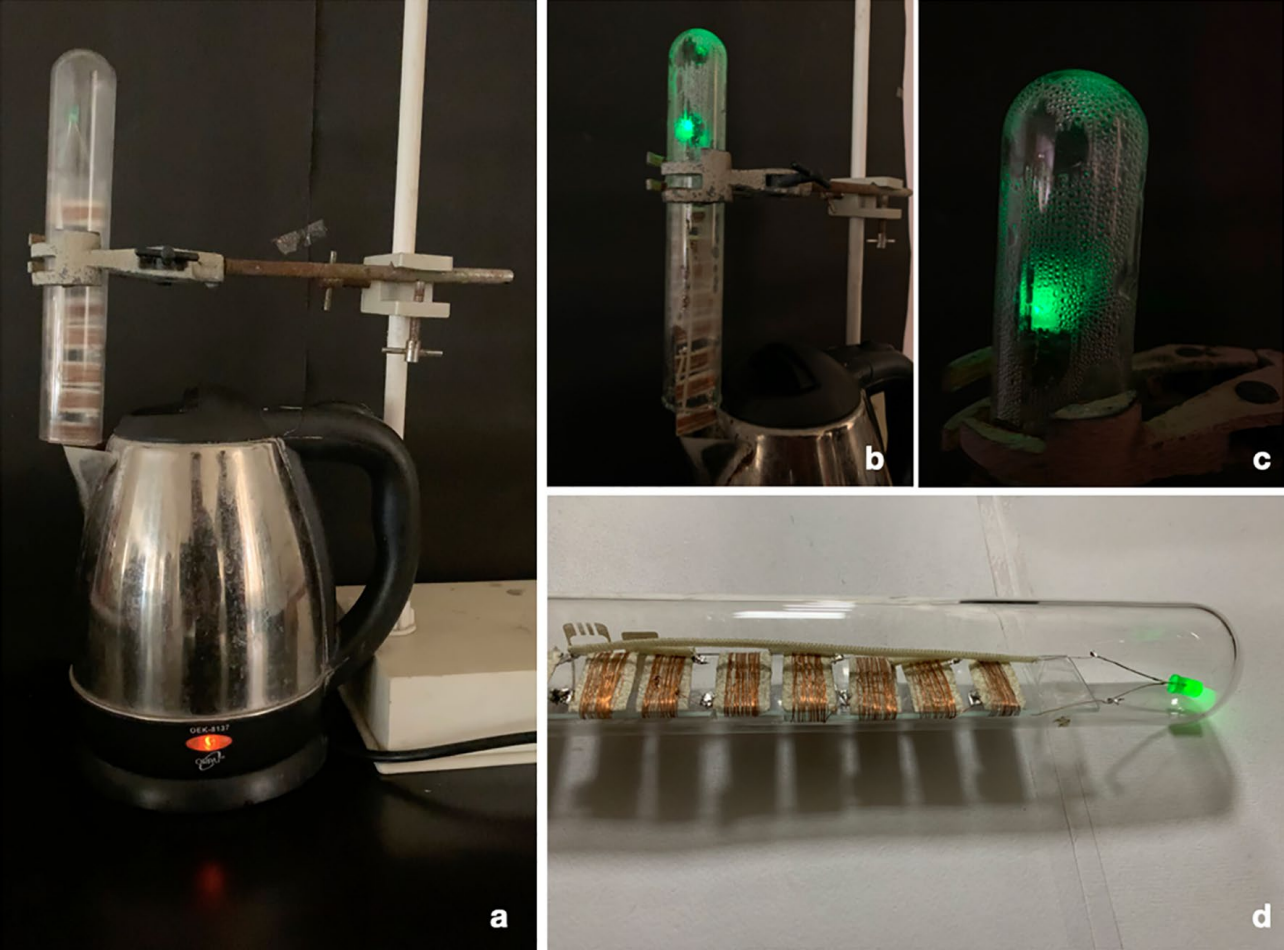
The self-lighting kettle. (**a**) The set-up shows the device at the mouth of the kettle. (**b**) Glowing LED after exposure to the water vapor. (**c**) A close view of the glowing LED and the condensation of the water vapor. (**d**) The device, after removal from the water vapor source.

**Figure 8:**
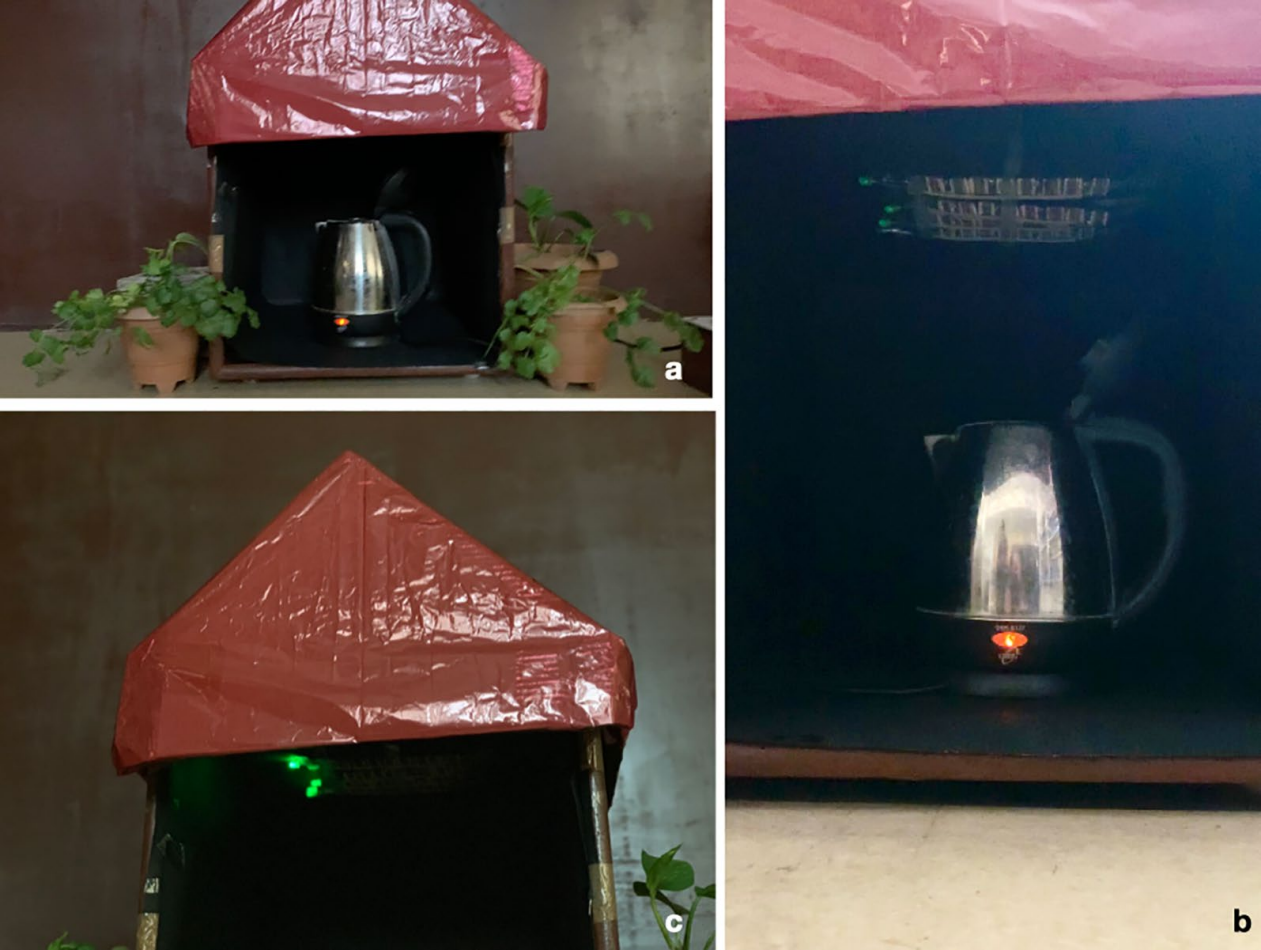
The water–vapor panels for futuristic homes. (**a**) The set-up shows the prototype of a model home with the panels made up of 'brine-silk cocoon protein bio-battery.' (**b**) The panels receive water vapor from the bottom. The glowing devices as seen on the roof. (**c**) The brightly glowing devices on the roof of the model house.

In the first device, we propose that we can use the waste heat generated by a kettle to power the device (Fig. 7a–d, Supplementary Video S1). We place the device at the mouth of the kettle, and after the initial water vapor exposure for 2 min, we remove the device. The device output generates a low current for the next 24 h, as seen in the form of a glowing green light-emitting diode. Similarly, in a model prototype of a house, we placed an array of devices that gets water vapor exposure from the bottom (Fig. 8a–c, Supplementary Video S2). The array of lights continues to operate for a prolonged time. These prototypes are examples of utilizing the waste heat to generate usable electricity.

## Discussion

The basic concept and fundamental mechanism of silk biobattery have its root in the asymmetric porous architecture of silk cocoons. The size of molecular pores is prominent at the outside surface and narrows as it approaches the inner surface. The cross-section of the pores within the silk cocoon membrane resembles a tapered pipe-like geometry, which narrows towards the inner side. Our earlier findings have shown that water takes significantly more time to enter the silk cocoon than when it comes from inside to outside. In a sense, the pore asymmetry makes the silk cocoon membrane a waterproof structure^9–11^. So, if all the pores fill with water, the silk membrane will have a water gradient, resulting in a potential difference^10,11^. Suppose we apply heat to this water-filled structure, then the water, along with the side chains of the silk membrane protein, experiences molecular motion. At this stage, the polaron-like and proton (H_3_O^+^) moieties generate and function as charge carriers along the asymmetric porous pipes of the silk cocoon membrane^10,11,16^. The silk cocoon membrane's low inherent potential difference offers slight directionality to these polaron-like charge carriers^10,11,16^. So we create an additional potential difference by placing the membrane between asymmetric electrodes with different electronegativity values. We chose aluminum and copper with electronegativity values of is 1.61 and 1.90, respectively, using the Pauling scale.

**Figure 9:**
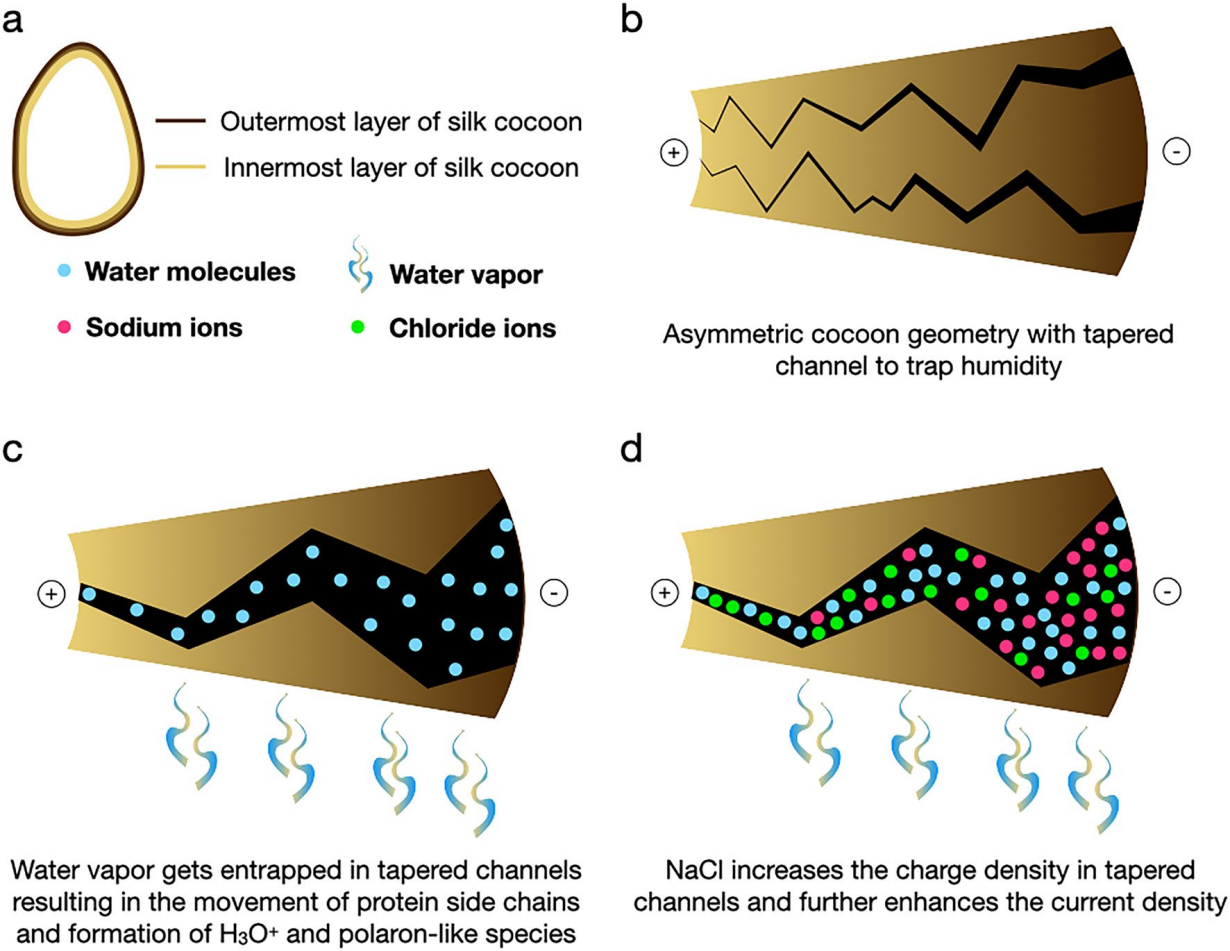
Putative mechanism of silk cocoon biobattery. (**a**) The picture of the cocoon shows the cocoon's distinct inner and outer surface to explain the asymmetric nature of the silk cocoon membrane. (**b**) The pores across the membrane are formed in such a way that it appears like tortuous, tapered channels. The channels narrow towards the inner surface and become more prominent at the outer surface. (**c**) When exposed to water vapor, the channels trap the water vapor and create a water gradient. Further heat causes movement in the proteins' side chains, which interact with the water molecules resulting in the formation of polaron-like complexes and H_3_O^+^ species. (**d**) The NaCl addition causes an increase in the density of the charge carriers and results in a significant increase in conductance.

Yet the challenge remains, how to increase the charge carriers. The charge carrier density will decide the current output. So, we enhance the charge carriers by imbibing the silk cocoon in a brine solution—the pores fill with sodium and chloride ions and water molecules. Now the system consists of an asymmetric silk membrane rich in ionic charge carriers, along with water molecules to assist the flow of ions. Figure 9 provides a graphical summary of the mechanism of silk biobattery.

To our surprise, the 2-min exposure to moist heat alters the surface properties of the silk cocoon membrane. The first event we observe (Table 1) is the proportionally more prominent exposure of the electron-dense, high-energy carbon species (C–N, C=C, C=O) on the cocoon surface. The second aspect (Table 1) we find is that the sodium and chloride ions penetrate the matrix. Since the silk cocoon system has an inherent asymmetry, the movement of charges within the matrix results in an asymmetric distribution following water vapor exposure. Most possibly, the ionic species interacting with protein chains and water forms clusters of ionic bridges resulting in enhanced conduction.

Further, the alternating 'water vapor–dry air' cycle causes rapid movement of water molecules across the asymmetric porous pipes of the silk cocoon membrane. This process results in quick recharging of the membrane. Otherwise, the membrane must partly dry out before we can recharge it. The critical point that emerges from this new silk-biobattery class is the optimal water concentration within the asymmetric silk cocoon membrane, sufficient charge carriers to generate adequate current and a swift perturbation of the system with heat.

The present study opens a few exciting aspects about the electrical properties of the silk cocoon protein membrane. The initial sharp rise in the current from the baseline post-exposure to water vapor resembles semiconductor-like features in the proteins. While the known semiconductors are mostly inorganic or organic, current results hint toward a research opportunity for protein-based semiconductor devices. With the advancement in biotechnology, the large-scale production of proteins is quite a feasible option^17–20^. We may find industrial applications for biodegradable, water-based, and flexible electronics in the future.

The next observation is the upward shift in baseline current after exposure to water vapor. The conductivity of the membrane improves after 2 min of exposure to water vapor, and it appears like a change in the state of conductance of the membrane-a somewhat similar phenomenon to memory acquisition by long-term potentiation in the neuronal network^21^. Once the wet membrane is thermally perturbed to a critical point, it triggers a cascade of electrical activity. The multiple charging-discharging cycles hint that the silk membrane has an inbuilt memory and gets active by moisture and heat. In essence, it is a water-based thermistor device.

The conductance further improved when the sodium and chloride ions percolated inside the protein matrix and possibly formed salt bridges. These nanoscopic salt bridges help in charge hopping across the cocoon protein. The most striking aspect is the persistence of the current in NaCl soaked cocoon exposed to water vapor. It is something that we observe for the first time-the cocoon protein function like a solid electrolyte matrix that reprograms in response to moisture and heat.

**Figure 10:**
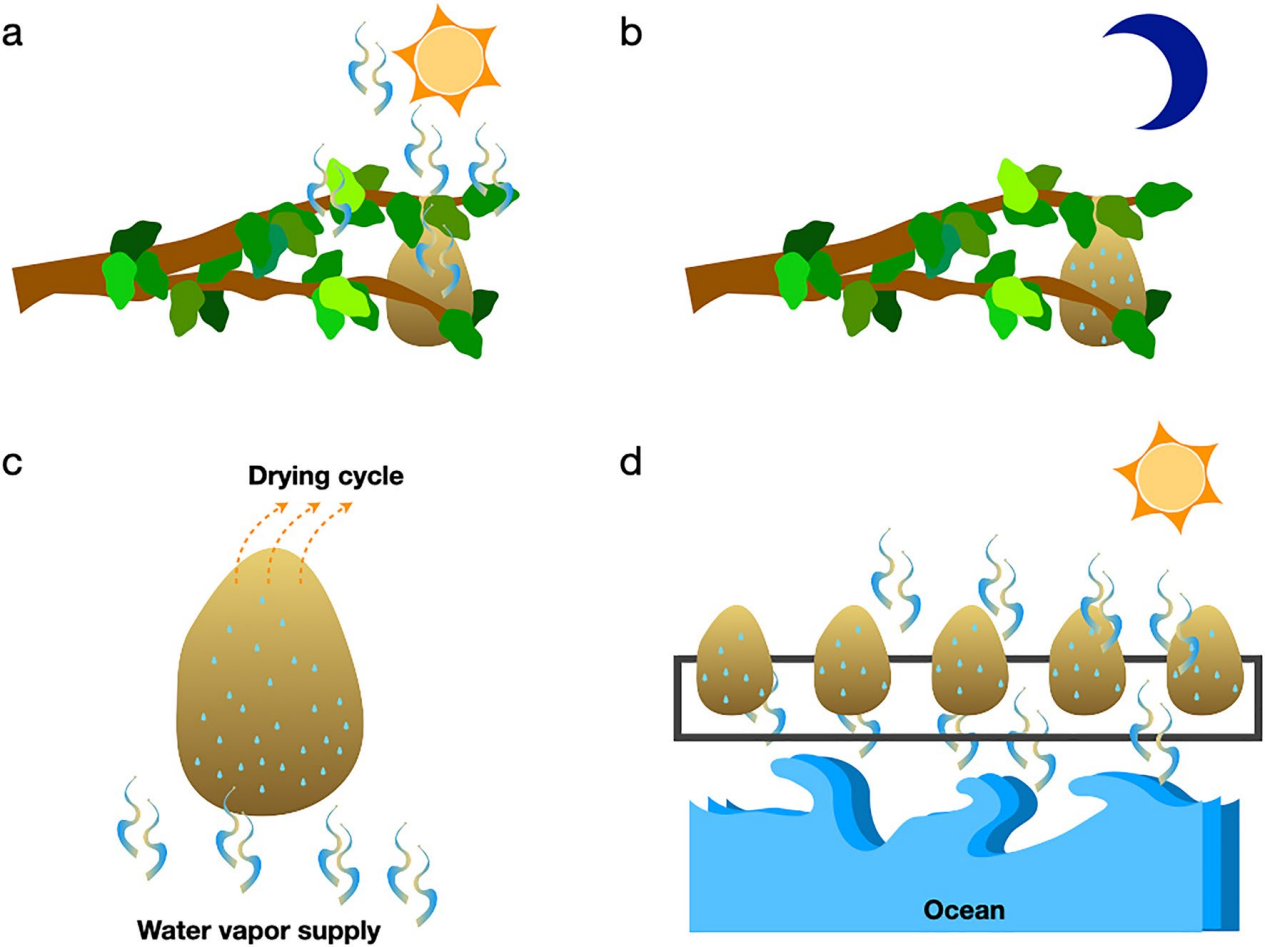
Cocoon ecology. (**a**) During day time, solar radiation causes evapotranspiration from nature. The humidity gets trapped in the silk cocoon's microstructured channels due to its proximity to the surrounding vegetation. (**b**) During the nighttime, the trapped moisture condenses inside the silk cocoon pores as the temperature falls. (**c**) The simultaneous application of dry air and water vapor causes a water gradient across the cocoon. (**d**) Cocoon's natural ability to trap moisture and convert it into green electricity makes it a suitable material for AWG devices.

The 'water vapor–dry air' cycle for rapid charging and discharging of the cocoon battery draws inspiration from cocoon ecology. The cocoon remains in a micro-ecosystem where the plant leaves offer it a moist ecosystem while the sunlight causes transpiration. So, like a thermocouple, the cocoon experiences a low and high-temperature regime. An optimal water level within the cocoon's pores helps support the growing worm inside it. The temperature and humidity are high, especially in the tropics, when the pupa emerges as a butterfly after metamorphosis^5^. So, the membrane is electrically charged to signal the butterfly to emerge from the cocoon^10,12^. We borrowed this idea from the cocoon biosystem and exposed it to an alternate 'water vapor–dry air' cycle to derive maximum current for a prolonged time. It is an example of a 'protein thermocouple' in some sense. Figure 10a–c explains the charging-discharging process in terms of cocoon ecology.

The evidence from XPS analysis throws light on some of these surface molecular events causing the barrage of electrical activities. The water vapor reduces the surface presence of low-energy carbon species (C–C, C–H). In contrast, the electron-dense, high-energy carbon species (C–N, C=C, C=O) remained unchanged, possibly enhancing surface charge hopping.

The fundamental difference between the conventional battery and silk cocoon biobattery is the charging with water vapor and exploiting the native silk cocoon membrane's asymmetric geometry. To date, no battery has the potential to get charged with water vapor. Atmospheric water vapor is an abundant, unlimited resource fuelled by the earth's hydrologic cycle. Three thousand one hundred cubic miles of the atmosphere is water vapor^22^. Tapping water vapor to generate electricity using a naturally occurring protein interface is the invention of this study. Further, the large ocean cover makes NaCl an abundant resource. At this stage, the current research reports a human-made prototype. In the future, when the technology will translate at an industrial scale, it holds the promise of tapping green electricity from all the earth's abundant natural resources. In the figure, we have given a proposed schematic for future application.

The ongoing quest to meet the demand for freshwater draws interest toward intense improvements in designing a more efficient Atmospheric Water Generator (AWG)^22^. The asymmetric geometry of silk cocoons, their natural tendency to trap humidity, and their ability to generate current may find a component innovation in the futuristic AWG development **(**Fig. 10d). With technological advancements in green manufacturing, designing a cocoon-inspired biopolymer architecture is an eco-friendly solution for humidity trapping material. The added inbuilt functionality to harvest electricity from such structures is of immense significance for meeting the energy needs in remote places, strategic locations, and the plethora of other unexplored aspects of engineering.

Apart from our efforts towards coupling the energy system with different water vapor releasing modules, we are working in the following areas. We are currently tweaking the system for food biosensor applications, integrating the system with the human body for low current therapies for chronic neurological problems. We are combining the design with an automobile exhaust pipe where a significant amount of water vapor condenses. The coupling of the current invention will be an additional mode to generate power from the automobile exhaust system.

Regarding the current study's limitations, we still need to explore the exact molecular events orchestrating the high conductance within the protein molecule. Nevertheless, the most promising aspect is that the present findings compel us to revise our bio-design perspective of manufacturing. Modern manufacturing processes struggle with excessive greenhouse gas emissions^23^. When considering proteins like silk, nature has optimized the sustainable manufacturing route through an evolutionary time scale. Other classic examples in nature's armoire of self-sustaining energy harvesting and storage systems are photo-sensitive chloroplast systems and mitochondria' electron-transport chains. The plethora of embedded proteins in these systems and other cells generate low-intensity wet currents while sensing light, heat, vibrations, smell, voltage, osmotic pressure, and pH. These proteins are channels, pumps, and pores crucial to our brain, heart, and muscle functioning. The biosystems rely on these minuscule amounts of wet electricity generated by these proteins throughout their survival. Nature has optimized its genetic and enzymatic machinery to minimize the carbon footprint in producing these proteins. However, the technological challenge is to isolate these proteins while maintaining their functional integrity. Further, the challenge is how to increase the current output.

In the present study, we borrow the intelligence of the silk cocoon worm. Silkworms develop this wet thermoelectric material-silk cocoon to orchestrate metamorphosis. We borrow the inbuilt intelligence of this robust protein and increase its charge carriers. The results are promising and open avenues for industrial-scale development of protein-based semiconductors, energy devices, incubators, drug carriers, and protein-electrodes for biomedical and bioelectronics applications.

## Supplementary Information

Supplementary Video S1.

Supplementary Video S2.

Supplementary Tables.

Supplementary Information.

## Data Availability

Any data used to generate the figures and support the text of this review are available on request made by email to the corresponding author: mainakd@iitk.ac.in, himanshi.jangir@ucf.edu.
