# Supplementary material for: Designing water vapor fuelled brine-silk cocoon protein bio-battery for a self-lighting kettle and water-vapor panels
Source: Sci Rep. 2022 Aug 17;12:13999. doi: 10.1038/s41598-022-18211-x (PMC9385712; doi:10.1038/s41598-022-18211-x)
Supplement: Supplementary file 3 — Supplementary Tables. [file 41598_2022_18211_MOESM3_ESM.docx]

**Supplementary information**

**Designing water vapor fuelled brine-silk cocoon protein bio-battery for a self-lighting kettle and water-vapor panels**

Himanshi Jangir**^1*^**, Mainak Das**^2*^**

**^1^**NanoScience Technology Center, University of Central Florida, Orlando, Fl, 32826, USA

**^2^**Design Department, Indian Institute of Technology Kanpur, Kanpur, UP, 208016, India

*****Corresponding authors. HJ and MD are co-corresponding authors

Email: [himanshi.jangir@ucf.edu](mailto:himanshi.jangir@ucf.edu) or [mainakd@iitk.ac.in](mailto:mainakd@iitk.ac.in)

In the supplementary section, we have a video **(Video DIY)** explaining the process of making the water vapor-fuelled brine-silk cocoon protein bio-battery.

**Table 1.** Dose optimization for NaCl. The third row (12.5 g NaCl) shows the highest current output among the three devices.

| NaCl solution soaking for 24 hours | Molarity | Average peak current  n= number of devices |
| --- | --- | --- |
| 0.875 g NaCl in 25 ml double distilled water (598 mM) used to soak 16 cocoon pieces to prepare 2 devices. | 598 mM  Approximately sea water concentration of NaCl | 2.3 mA  n=4 |
| 6.125 g NaCl in 25 ml double distilled water used to soak 16 cocoon pieces to prepare 2 devices. | ~4.19 M | 2.5 mA  n=6 |
| 12.5 g NaCl in 25 ml double distilled water used to soak 16 cocoon pieces to prepare 2 devices. | ~8.58 M | 3.6 mA  n=12 |

**Distill water, NaCl, and KCl devices:** Like we soaked the silk cocoon in brine, we soaked them in potassium chloride (KCl). The **table 2** below compares the electrical data obtained from distilled water, NaCl, and KCl devices. After comparing the current values, we opted for NaCl devices for our silk cocoon biobattery.

**Table 2.** We compared the current generated by distilled water, NaCl, and KCl devices. The second row (NaCl) shows the highest current output among the three devices.

| Devices | Average baseline current  ±standard error;  n= number of  devices | Average peak current  ±standard error;  n= number of devices | Average current after 1 hour  ± standard error;  n= number of  devices |
| --- | --- | --- | --- |
| Distilled water | 9.33e-06±6.24e-06 mA; n=6 | 0.014±0.003 mA; n=6 | 0.002±0.0001 mA;  n=6 |
| NaCl | 0.06±0.02 mA;  n=12 | 3.63±1.07 mA;  n=12 | 0.39±0.10 mA;  n=12 |
| KCl | 0.005±0.004 mA;  n=6 | 0.86±0.26 mA;  n=6 | 0.07±0.02 mA;  n=6 |
